# Biomimetic ZIF-8 Nanoplatform for Enhanced Therapeutic Efficacy of Combined Phototherapy and Chemotherapy Against Hepatocellular Carcinoma

**DOI:** 10.3390/pharmaceutics18081000

**Published:** 2026-08-13

**Authors:** Xinlei Lin, Shaoteng Huang, Ning Zheng, Wenjie Yao, Mingbo Zhang, Qingqing Tu, Longhua Shen, Tao Wang, Gang Niu, Fang Wang, Junyang Zhuang, Yang Chen, Ning Li

**Affiliations:** 1 Department of Hepatobiliary Surgery, Fuzhou Second General Hospital, Fuzhou 350007, China; 2 Fujian Key Laboratory of Drug Target Discovery and Structural and Functional Research, School of Pharmacy, Fujian Medical University, Fuzhou 350122, Chinajunyang.zhuang@hotmail.com (J.Z.); 3 Fujian Key Laboratory of Natural Medicine Pharmacology, Department of Pharmacology, School of Pharmacy, Fujian Medical University, Fuzhou 350122, China; 4 Department of Oral and Maxillofacial Surgery, School and Hospital of Stomatology, Fujian Medical University, Fuzhou 350002, China

**Keywords:** biomimetic nanoplatform, combination therapy, cell membrane camouflage, phototherapy, nanomedicine

## Abstract

**Background**: Hepatocellular carcinoma (HCC) remains challenging to treat because of the limited therapeutic efficacy and insufficient selectivity of conventional therapies. To overcome these limitations, multifunctional nanoplatforms integrating biomimetic strategies and combination therapy have attracted increasing attention. Single-modality therapies are often limited by insufficient therapeutic efficacy and restricted mechanisms of action, highlighting the need for biomimetic nanoplatforms that integrate combination therapeutic strategies for enhanced antitumor performance. **Methods**: Herein, a biomimetic strategy-based nanoplatform (DI-ZM) was constructed via a combination of ZIF-8 biomineralization, physical adsorption of dihydroartemisinin (DHA) and indocyanine green (ICG), followed by HepG2 cell membrane coating to achieve homologous interaction. This design enables integrated chemotherapy, photothermal therapy (PTT), and photodynamic therapy (PDT) within a single system. **Results**: The resulting DI-ZM nanoparticles exhibited a hydrodynamic diameter of approximately ~200 nm with good colloidal stability and high drug-loading capacity. Under 808 nm laser irradiation, DI-ZM achieved a temperature elevation to ~66 °C within 5 min, together with efficient ROS generation. Compared with uncoated nanoparticles, the biomimetic membrane coating significantly enhanced cellular uptake and homologous targeting ability, as confirmed by CLSM and flow cytometry analysis. Benefiting from the biomimetic membrane coating, DI-ZM further exhibited improved homologous targeting and cellular uptake, which contributed to enhanced intracellular ROS generation. This was accompanied by significant mitochondrial membrane depolarization and apoptosis rates exceeding 80% in HepG2 cells under laser irradiation, ultimately resulting in markedly enhanced cytotoxicity. In addition, the biomimetic membrane coating also enabled efficient penetration of DI-ZM into multicellular tumor spheroids, indicating its improved tumor-penetration capability. In vivo antitumor studies further revealed effective tumor suppression with a tumor inhibition rate of approximately 97%, along with acceptable systemic tolerance in HepG2 tumor-bearing mice. **Conclusion**: The biomimetic membrane-coated ZIF-8 nanoplatform integrating chemotherapy with ICG-mediated phototherapy (photothermal and photodynamic therapy) provides an effective strategy for the combination therapy against HCC.

## 1. Introduction

Hepatocellular carcinoma (HCC) remains a major challenge in clinical treatment because of the limited therapeutic efficacy and insufficient selectivity of conventional therapies [1]. Although chemotherapy is widely used for HCC treatment, free anticancer drugs often suffer from poor stability, insufficient intracellular delivery, and undesirable side effects, which significantly restrict their therapeutic performance [2,3]. Recently, increasing attention has been directed toward the repositioning of conventional drugs for anticancer applications [4]. Dihydroartemisinin (DHA), a semisynthetic derivative of artemisinin originally developed as an antimalarial agent [5], has demonstrated considerable anticancer activity in various tumor models through ROS-associated oxidative stress and apoptosis induction, providing a promising alternative for cancer chemotherapy [6,7,8].

In recent years, photothermal therapy (PTT) has attracted increasing attention as a minimally invasive therapeutic strategy owing to its favorable spatiotemporal controllability and therapeutic potential [9,10,11,12]. Indocyanine green (ICG), an FDA-approved near-infrared dye, has been extensively investigated in tumor phototherapy because of its photothermal and photodynamic properties [13]. Upon laser irradiation, ICG can convert near-infrared light into heat while simultaneously generating reactive oxygen species (ROS), thereby enabling combined photothermal and photodynamic therapeutic effects [14,15,16,17]. However, the therapeutic efficacy of free ICG is still limited by poor photostability, insufficient intracellular delivery, and inadequate tumor penetration [18,19]. In addition, monotherapies often fail to achieve satisfactory therapeutic outcomes because of their single therapeutic modality [20,21,22]. Therefore, the development of multifunctional nanoplatforms capable of integrating efficient drug delivery with multimodal therapy is highly desirable for enhanced HCC treatment [23,24,25].

Nanotechnology provides an effective strategy to improve the therapeutic performance of anticancer agents by enhancing drug stability, intracellular delivery, and tumor retention [26,27,28,29,30,31,32]. Previous studies have explored various nanoplatforms for chemo-phototherapy, including polymeric nanoparticles, lipid-based carriers, inorganic nanoparticles, and metal–organic framework (MOF)-based systems. These platforms have shown advantages in improving drug solubility, protecting photosensitizers, and enabling the co-delivery of chemotherapeutic and phototherapeutic agents. However, many conventional systems still rely mainly on passive accumulation and may show limited tumor-cell-specific uptake or insufficient penetration within compact tumor tissues.

Among these systems, zeolitic imidazolate framework-8 (ZIF-8), a representative MOF material, has attracted considerable attention because of its high drug-loading capability, thermal stability, favorable biocompatibility, and pH-responsive degradation behavior [33,34,35,36]. These characteristics make ZIF-8 an attractive carrier for constructing pH-responsive co-delivery systems. Nevertheless, unmodified ZIF-8 nanoparticles generally lack tumor-cell-specific recognition and may still show limited cellular uptake and tumor penetration [37,38]. Recently, biomimetic strategies have emerged as promising approaches for improving the biological performance of nanomedicines [39,40,41,42,43]. Among these strategies, cell membrane coating has attracted considerable attention because it enables synthetic nanocarriers to retain selected biological characteristics of the source cells while preserving the physicochemical functions of the underlying core. The resulting biological behavior is closely related to the origin of the membrane. Red blood cell membranes are widely used to reduce immune clearance and prolong systemic circulation; however, they generally lack intrinsic tumor-cell-specific recognition and often require additional surface functionalization to achieve active tumor targeting. Leukocyte- and macrophage-derived membranes can exploit natural inflammatory recruitment and transendothelial migration pathways, but their biological functions may vary with the cell subtype, activation state, and membrane preparation process. In contrast, cancer cell membranes retain a broad spectrum of source-cell surface components involved in cell adhesion and self-recognition, providing a natural basis for homotypic interactions and preferential uptake by cancer cells of the same origin [44,45,46]. Therefore, coating DHA/ICG-loaded ZIF-8 nanoparticles with HepG2 cell membranes may integrate the pH-responsive delivery properties of the MOF core with a biomimetic interface capable of enhancing homologous cellular recognition, cellular uptake, and penetration into multicellular tumor spheroids.

**Scheme 1:**
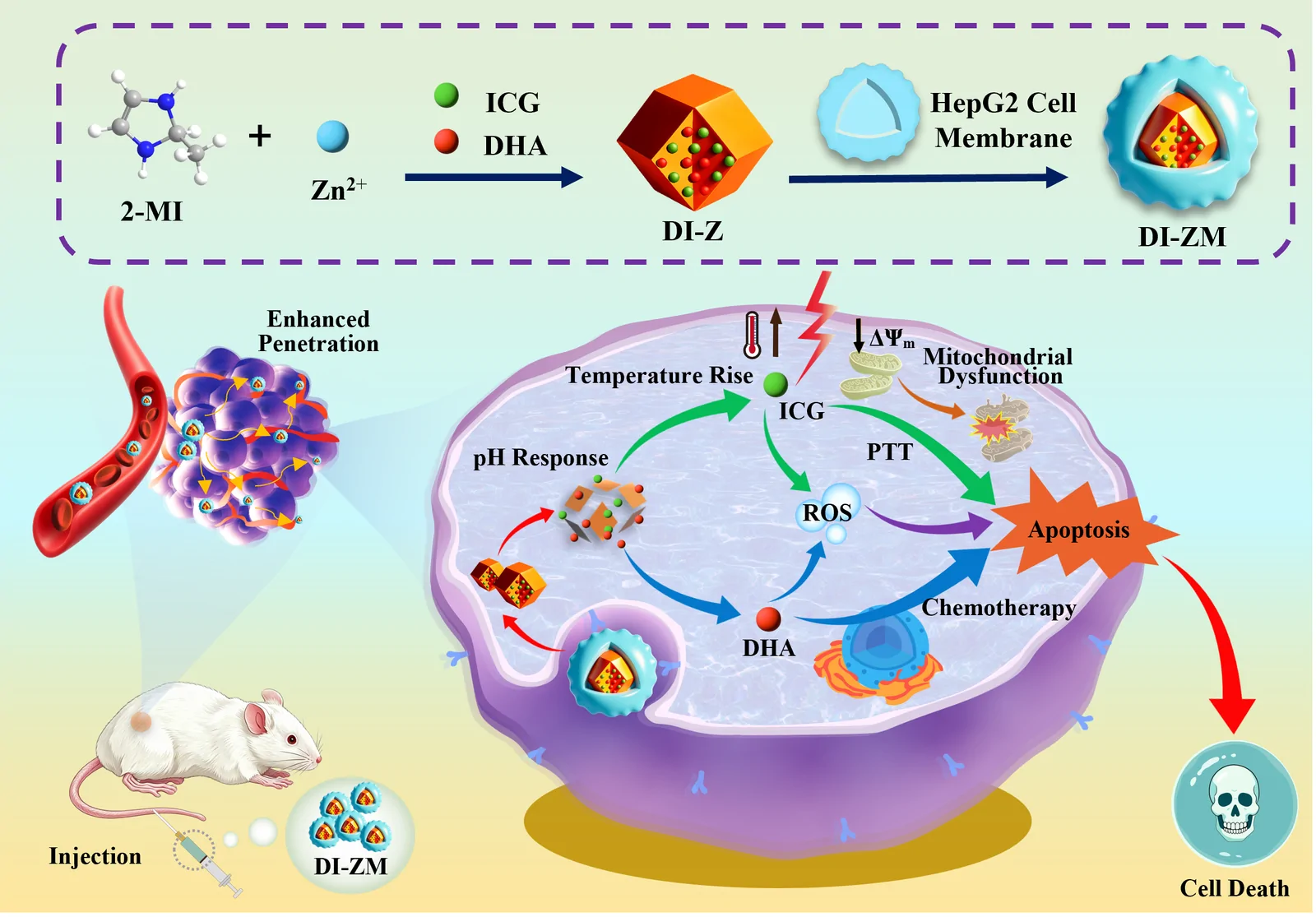
Schematic illustration of a biomimetic drug delivery system for combined chemotherapy and phototherapy against Hepatocellular carcinoma.

Based on these considerations, we developed a biomimetic HepG2 cell membrane-coated ZIF-8 nanoplatform (DI-ZM) for the co-delivery of DHA and ICG, as displayed in Scheme 1. Specifically, ZIF-8 nanoparticles were first constructed through biomineralization and subsequently loaded with DHA and ICG via physical adsorption, followed by coating with HepG2 cell membranes to obtain the biomimetic DI-ZM nanoplatform. The integration of DHA-mediated chemotherapy with ICG-based phototherapy was expected to provide enhanced therapeutic efficacy against HCC through multimodal therapeutic effects. The obtained DI-ZM nanoparticles were designed to combine the advantages of biomimetic targeting, enhanced intracellular delivery, improved tumor spheroid penetration, and multimodal therapeutic effects. The physicochemical properties, photothermal behavior, ROS generation capability, homologous targeting ability, and multicellular tumor spheroid penetration of DI-ZM were systematically investigated. In addition, the therapeutic efficacy of DI-ZM was evaluated through intracellular ROS detection, mitochondrial membrane potential analysis, apoptosis assessment, and in vivo antitumor studies. This work provides an effective strategy for biomimetic nanotechnology-enabled combinational HCC therapy, suggesting the potential of biomimetic nanoplatforms for multimodal anticancer therapy.

## 2. Materials and Methods

### 2.1. Materials

Fetal bovine serum (FBS) was from Umedium (Hefei, China). The JC-1 Mitochondrial Membrane Potential (MitoMP) Detection Kit was purchased from Titan Scientific Co., Ltd. (Adamas-beta, Shanghai, China). The Annexin V-FITC/PI Apoptosis Detection Kit was purchased from Dojindo (Kumamoto, Japan). The matrigel was bought from Mongengel (Xiamen, China).

### 2.2. Methods

#### 2.2.1. Preparation of Biomimetic Nanoparticles

Indocyanine green (ICG, 1.5 mg) was dissolved in 2-methylimidazole solution (3 mL, 7.5 M). Zinc nitrate solution (0.5 M, 120 μL) was then introduced dropwise under continuous stirring and the reaction was allowed to proceed for 20 min at room temperature. The resulting nanoparticles were collected by centrifugation (12,000 rpm, 4 °C, 15 min) and washed twice with ultrapure water to remove residual reactants, yielding ICG@ZIF-8.

Dihydroartemisinin (DHA, 2.4 mg) was dissolved in an ethanol–water mixture and maintained at 50 °C. The ICG@ZIF-8 suspension was subsequently added and stirred for 4 h. After purification by centrifugation and washing, DHA&ICG@ZIF-8 (abbreviated as DI-Z) was obtained.

DI-Z was combined with HepG2 cell membranes, which were prepared in advance using a commercial membrane extraction kit (Invent Biotechnologies, Inc., Beijing, China) according to the supplier’s protocol, and incubated at 4 °C, followed by sonication in an ice bath to generate membrane-coated nanoparticles, yielding DHA&ICG@ZIF-8@HepG2 cell Membrane (DI-ZM).

#### 2.2.2. Particle Size, Zeta Potential, and Morphology

Samples were diluted with ultrapure water prior to measurement. Hydrodynamic diameter and zeta potential were measured using dynamic light scattering (Anton Paar Group AG, Graz, Austria). Meanwhile, morphology was further observed by transmission electron microscopy (FEI Company, Hillsboro, OR, USA).

#### 2.2.3. Determination of Drug Loading Efficiency

The loading content and encapsulation efficiency of ICG and DHA were quantified by UV–vis spectroscopy. Supernatants collected during nanoparticle preparation were freeze-dried and redissolved in DMSO for quantification of unencapsulated drugs. The concentration of ICG was determined by measuring the absorbance at 795 nm, while DHA was quantified at 238 nm after reaction with 2% NaOH at 60 °C for 30 min. Encapsulation efficiency and drug loading were calculated accordingly.

#### 2.2.4. Cellular Uptake Study

HepG2 cells were seeded in glass-bottom dishes at a density of 1 × 10^5^ cells per dish and cultured for 12 h. Cells were then incubated with DI-Z or DI-ZM at an ICG-equivalent concentration of 5 μg/mL for 2, 4, 6, or 8 h. Following incubation, cells were washed three times with PBS, fixed with 4% paraformaldehyde for 15 min, and stained with DAPI for 5 min. After washing, cells were imaged using CLSM. ICG excitation wavelength 780 nm, emission wavelength 850 nm.

#### 2.2.5. Homologous Targeting Study

HepG2 (human hepatocellular carcinoma), 4T1 (murine breast cancer), SCC7 (murine squamous cell carcinoma), and Cal27 (human oral squamous carcinoma) cells were seeded in glass-bottom dishes (1 × 10^5^ cells per dish). After 12 h, the medium was replaced with DI-ZM-containing medium (ICG concentration: 5 μg/mL). After 4 h of incubation, the medium was removed and cells were washed thrice with PBS. Cells were subsequently fixed with 4% paraformaldehyde for 15 min, followed by DAPI staining for 5 min. After washing, intracellular fluorescence was observed by CLSM.

#### 2.2.6. Multicellular Tumor Spheroid Penetration

HepG2 multicellular tumor spheroids were constructed using a methylcellulose-assisted hanging drop method, as previously reported by our group [29]. After 48 h of incubation, spheroids were transferred to agarose-coated plates and cultured for an additional week to reach maturation. Then, the spheroids were incubated with ICG, DI-Z, or DI-ZM at an ICG-equivalent concentration of 10 μg/mL for 4 h. The distribution of fluorescence within the spheroids was then observed using CLSM to evaluate their penetration behavior.

#### 2.2.7. Apoptosis Analysis

HepG2 cells were seeded in 6-well plates and incubated overnight. Cells were then incubated with free DHA, free ICG (+/−L), DI-Z (+/−L), or DI-ZM (+/−L), with DHA- and ICG-equivalent doses of 8 and 10 μg/mL, respectively. Cells in the irradiation groups (+L) were irradiated (808 nm, 0.75 W/cm^2^, 5 min) after 4 h of incubation, followed by a total incubation period of 24 h. Cells were subsequently collected, washed thrice with PBS, and resuspended in binding buffer. Annexin V-FITC and propidium iodide (PI) were added according to the manufacturer’s instructions, and samples were incubated for 15 min in the dark. Finally, samples were analyzed by flow cytometry (BD FACSCanto (TM) II flow cytometer, BD Biosciences, Franklin Lakes, NJ, USA). Annexin V-FITC: excitation wavelength 488 nm, emission wavelength 525 nm. PI: excitation wavelength 535 nm, emission wavelength 615 nm.

#### 2.2.8. In Vivo Antitumor Study

A subcutaneous HepG2 tumor model was established in nude mice. When tumor volumes reached approximately 100 mm^3^, mice were randomly assigned to six groups (*n* = 3 per group): PBS (control), free DHA, free ICG (+L), DI-Z (+L), DI-ZM (−L), and DI-ZM (+L). All formulations were administered via intravenous injection at doses equivalent to DHA (1.4 mg/kg) and ICG (2.0 mg/kg). At 3 h post-injection, mice in the irradiation groups (+L), including free ICG (+L), DI-Z (+L), and DI-ZM (+L), were irradiated (808 nm, 0.75 W/cm^2^, 5 min). Treatments were performed every two days for a total of three cycles. Tumor volume and body weight were monitored throughout the experiment. At the end of the study, mice were euthanized, and tumors were excised and weighed for further analysis.

#### 2.2.9. Statistical Analysis

All data are expressed as mean ± standard deviation (SD). Statistical analysis was performed using GraphPad Prism 8.0.2, and *p* < 0.05 was considered statistically significant. Statistical significance was denoted as * *p* < 0.05, ** *p* < 0.01, and *** *p* < 0.001.

## 3. Results

### 3.1. Preparation and Characterization of Biomimetic Nanoparticles

#### 3.1.1. The Size and Morphology Assays

**Figure 1:**
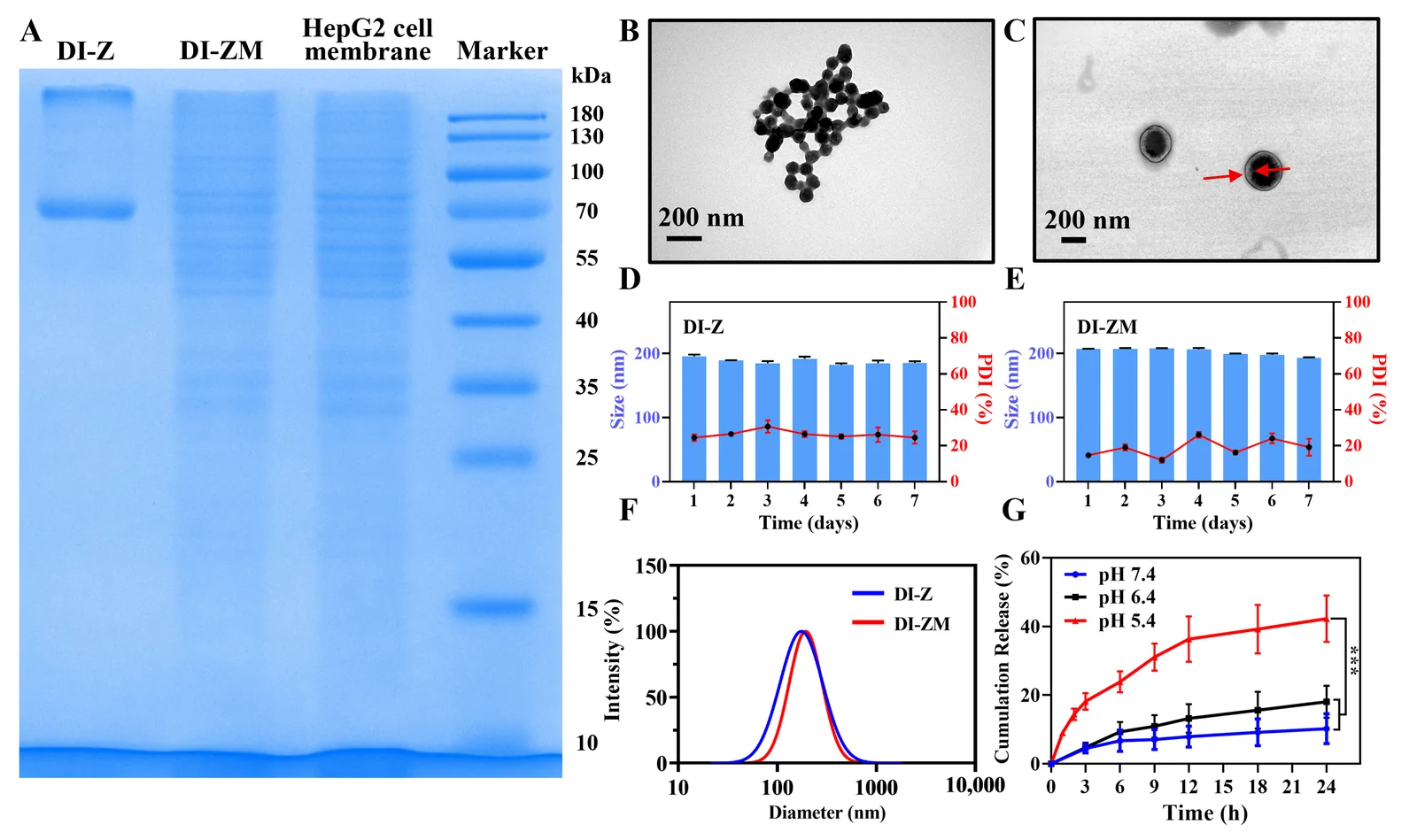
(**A**) SDS-PAGE analysis of HepG2 cell membrane proteins and membrane proteins retained on DI-ZM nanoparticles. TEM images of (**B**) DI-Z and (**C**) DI-ZM nanoparticles. Scale bars: 200 nm. The red arrows indicate the cell membrane coating surrounding the nanoparticle cores. Colloidal stability of (**D**) DI-Z and (**E**) DI-ZM nanoparticles over 7 days. (**F**) Hydrodynamic size distribution of DI-Z and DI-ZM nanoparticles. (**G**) pH-responsive release behavior of DHA from DI-ZM. *** *p* < 0.001.

The biomimetic nanoplatform DI-ZM was successfully constructed. SDS–PAGE profiles showed that DI-Z lacked characteristic membrane protein bands, whereas DI-ZM exhibited protein bands closely matching those of HepG2 cell membranes, confirming successful membrane coating (Figure 1A). TEM images further showed a clear core–shell structure for DI-ZM that was absent in DI-Z, supporting the formation of a membrane-coated architecture (Figure 1B,C).

Dynamic light scattering (DLS) analysis indicated that both DI-Z and DI-ZM exhibited uniform size distributions and good dispersibility, while the changes in hydrodynamic size further supported successful membrane coating (Figure 1F). DI-Z showed a hydrodynamic diameter of 195.7 ± 2.59 nm with a PDI of 0.20 ± 0.0185, whereas DI-ZM exhibited a slightly increased hydrodynamic diameter of 206.8 ± 0.46 nm and a PDI of 0.10 ± 0.0035. The increase in hydrodynamic size after membrane coating was consistent with the core–shell morphology observed by TEM. Notably, DI-ZM showed a more negative zeta potential, which is consistent with the presence of membrane components (Figure S1). Specifically, the zeta potential shifted from −20.20 ± 0.32 mV for DI-Z to −24.43 ± 0.43 mV for DI-ZM after membrane coating. In addition, DI-ZM displayed improved colloidal stability over seven days, with smaller fluctuations in particle size and PDI compared to DI-Z (Figure 1D,E), suggesting enhanced stability after membrane coating.

#### 3.1.2. Drug Loading Efficiency

UV–vis analysis confirmed the co-loading of the chemotherapeutic agent DHA and the photothermal/photodynamic agent ICG into the ZIF-8-based carrier. The encapsulation efficiencies of DHA and ICG were determined to be 29.44 ± 2.16% and 60.39 ± 0.22%, respectively. The corresponding loading capacities were 13.53 ± 0.76% and 19.95 ± 0.04%, respectively. Together, these results confirmed the successful incorporation of both therapeutic agents into the DI-ZM nanoplatform.

#### 3.1.3. Stability Studies

The colloidal stability of DI-Z and DI-ZM was evaluated over seven days. DI-Z maintained a relatively stable hydrodynamic diameter of approximately 180–195 nm in water, with PDI values around 0.25, indicating acceptable dispersion stability under aqueous conditions. After HepG2 membrane coating, DI-ZM showed a hydrodynamic diameter of approximately 195–205 nm in both water and serum-containing medium over seven days, with PDI values maintained around 0.20. These results indicate that DI-ZM preserved good size stability and dispersibility not only in water but also in a serum-containing environment. Together, these results indicate that the membrane-coated DI-ZM formulation remained colloidally stable in both aqueous and serum-containing environments over seven days.

### 3.2. pH-Responsive Drug Release Behavior

The drug release behavior of DI-ZM was evaluated under different pH conditions to investigate the release profiles of both DHA and ICG (Figure 1G and Figure S3). For DHA release, DI-ZM showed a relatively faster release tendency under acidic conditions than under neutral conditions. At pH 5.4, approximately 20% of DHA was released within 3 h, whereas only around 5% of DHA was released at pH 6.4 and pH 7.4 over the same period. During 24 h of incubation, the cumulative DHA release remained below 20% at pH 6.4 and below 10% at pH 7.4, indicating limited premature drug leakage under weakly acidic and physiological conditions. A similar pH-dependent release tendency was also observed for ICG, and the corresponding release data are provided in Figure S3. The relatively increased release under acidic conditions may be associated with partial destabilization of the ZIF-8 framework in tumor-relevant acidic environments, thereby facilitating payload liberation. Overall, these results indicate a pH-dependent release tendency of DI-ZM and suggest that acidic microenvironments may facilitate the release of the loaded agents from the ZIF-8-based nanoplatform.

### 3.3. Photothermal Performance Evaluation

**Figure 2:**
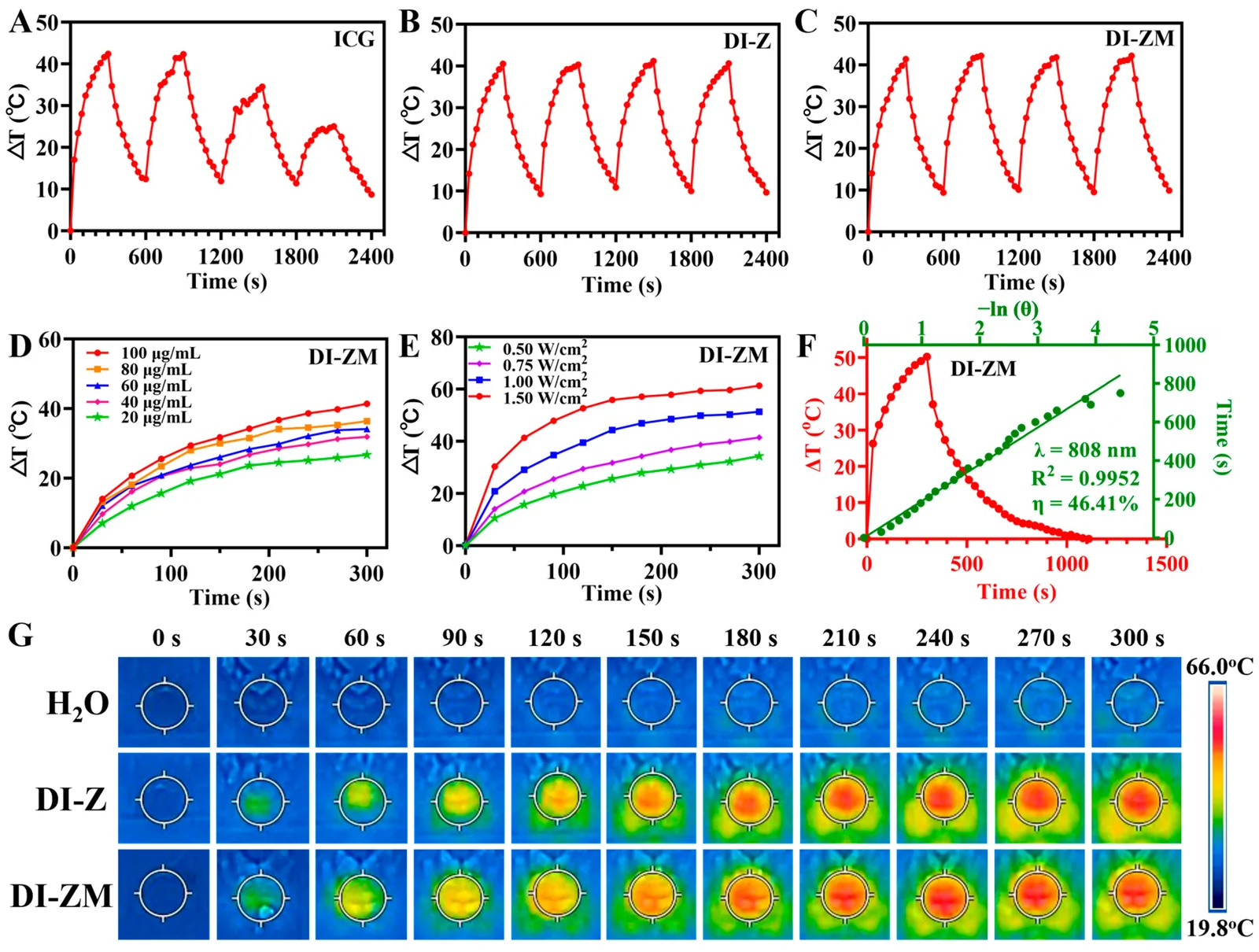
Evaluation of photothermal performance. Photothermal stability of (**A**) free ICG, (**B**) DI-Z, and (**C**) DI-ZM under four repeated NIR laser on/off irradiation cycles. (**D**) Photothermal heating curves of DI-ZM at different concentrations. (**E**) Photothermal heating curves of DI-ZM under different laser power densities. (**F**) Photothermal conversion efficiency of DI-ZM. (**G**) Infrared thermal images of H_2_O, DI-Z, and DI-ZM under laser irradiation (808 nm, 0.5 W/cm^2^, 5 min, 10 mm in diameter) at equivalent ICG concentration.

The photothermal performance of DI-Z and DI-ZM was evaluated under NIR laser irradiation. Compared to free ICG, both DI-Z and DI-ZM exhibited improved photothermal stability over four irradiation cycles, indicating enhanced photostability after encapsulation (Figure 2A–C). The temperature increase was positively correlated with both ICG concentration and laser power, demonstrating controllable photothermal behavior (Figure 2D,E). Notably, DI-Z and DI-ZM displayed higher photothermal conversion efficiencies (34.90% and 46.41%, respectively) than free ICG (33.72%), suggesting that the nanoparticle system improves the photothermal conversion efficiency of ICG (Figure 2F and Figure S4C,F). Infrared thermal imaging further confirmed the pronounced temperature elevation of DI-Z and DI-ZM under laser irradiation, whereas only negligible temperature changes were observed in the water group (Figure 2G). Notably, the temperature of the DI-ZM dispersion rapidly reached approximately 66 °C within 5 min of irradiation. These results indicate that the ZIF-8-based nanoparticles effectively maintain and improve the photothermal performance of ICG after encapsulation.

### 3.4. Reactive Oxygen Species (ROS) Generation

**Figure 3:**
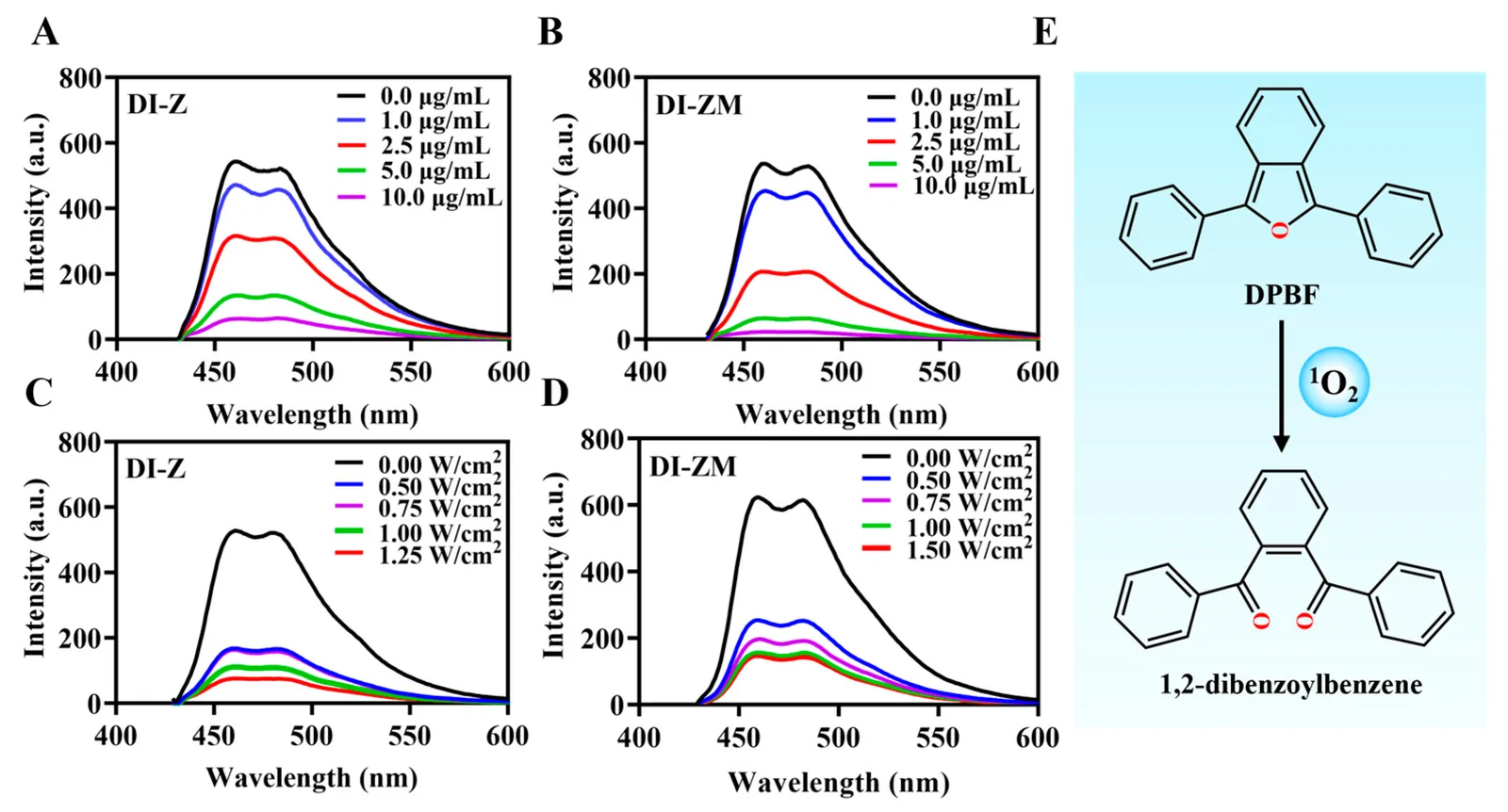
Evaluation of ROS generation capability. (**A**,**B**) Changes in DPBF absorbance induced by DI-Z and DI-ZM at different concentrations under laser irradiation. (**C**,**D**) Changes in DPBF absorbance induced by DI-Z and DI-ZM under different laser power densities. (**E**) Schematic illustration of singlet oxygen generation detected by DPBF.

Beyond photothermal effects, the ROS-generating capability of ICG was further evaluated. DPBF assays showed that both DI-Z and DI-ZM produced measurable amounts of ROS under laser irradiation, with ROS levels increasing with both ICG concentration and laser power (Figure 3A–D). These results indicate that the nanoparticles retain the ROS-generating capability of ICG. Together with the photothermal results, these findings suggest that the nanoparticles possess both photothermal and ROS-generating capabilities.

### 3.5. Cellular Uptake and Targeting Studies

**Figure 4:**
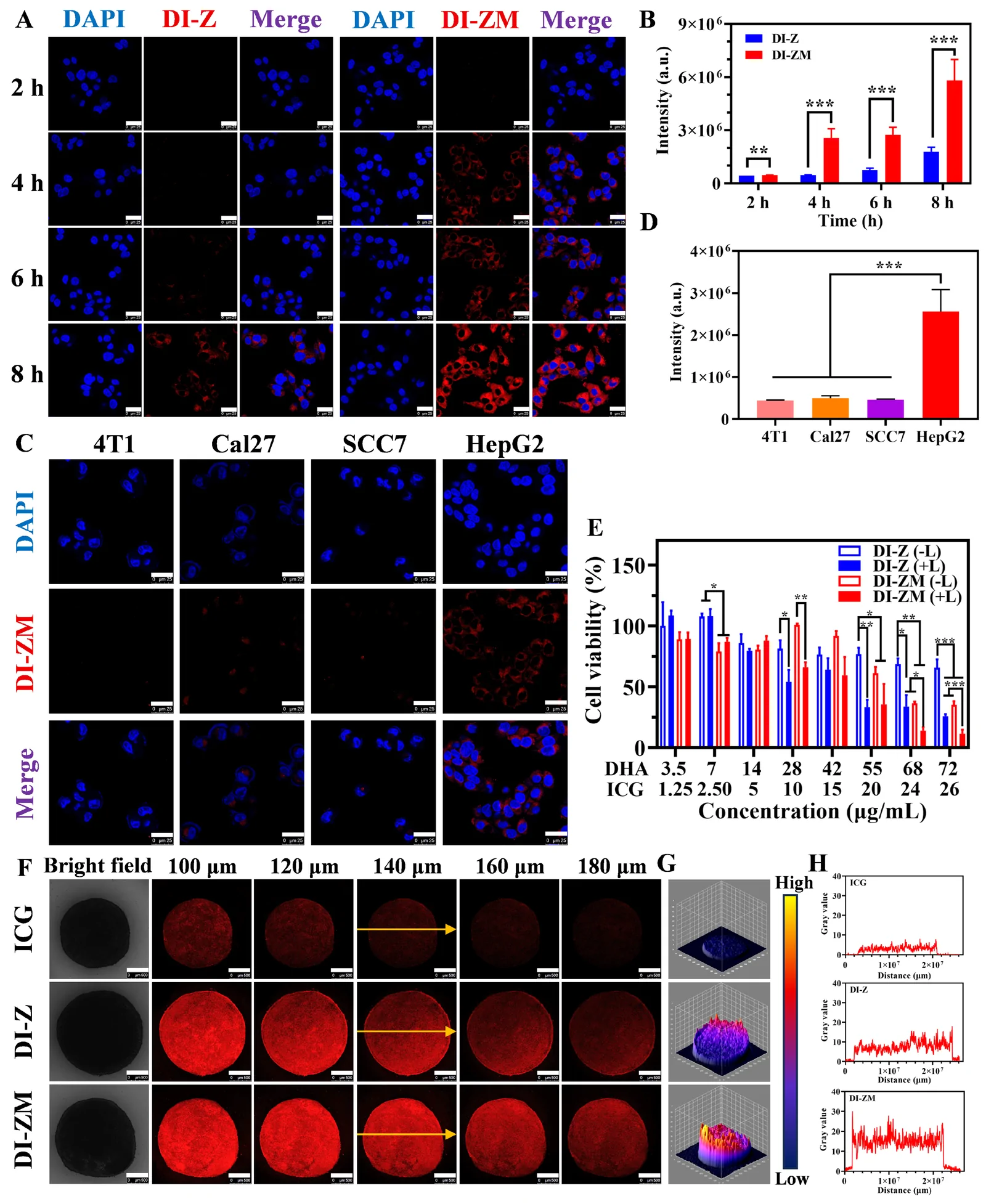
(**A**) CLSM images of cellular uptake of DI-Z and DI-ZM in HepG2 cells at various incubation times and (**B**) corresponding semi-quantitative fluorescence intensity analysis. Scale bars: 25 μm. (**C**) CLSM images of cellular uptake of DI-ZM in different cell lines and (**D**) corresponding semi-quantitative fluorescence intensity analysis. Scale bars: 25 μm. (**E**) MTT assay of the cytotoxicity of DI-Z and DI-ZM with or without laser irradiation toward HepG2 cells (n = 3). (**F**) CLSM images of the penetration behavior of free ICG, DI-Z, and DI-ZM in HepG2 multicellular tumor spheroids. (**G**) Three-dimensional reconstruction images of fluorescence distribution within tumor spheroids. (**H**) Fluorescence intensity profile analysis of spheroid penetration. Scale bars: 500 μm. * *p* < 0.05, ** *p* < 0.01, *** *p* < 0.001.

Cellular uptake of the nanoparticles was evaluated by CLSM together with semi-quantitative fluorescence analysis. As shown in Figure 4A,B, DI-ZM exhibited markedly stronger intracellular fluorescence signals than DI-Z at each time point. Notably, a clear intracellular signal for DI-ZM was detectable at 4 h, whereas DI-Z displayed negligible fluorescence and only showed a more pronounced signal at 8 h. Semi-quantitative analysis further confirmed significantly higher uptake of DI-ZM compared to DI-Z throughout the incubation period.

To evaluate homologous targeting, the uptake behavior of DI-ZM was further examined in different cell lines. As shown in Figure 4C,D, DI-ZM exhibited markedly higher fluorescence intensity in HepG2 cells compared to 4T1, Cal27, and SCC7 cells. Semi-quantitative analysis confirmed significantly higher uptake in HepG2 cells than in the non-homologous cell lines. The enhanced uptake in homologous cells suggests that membrane coating promotes preferential recognition and internalization.

To further verify the contribution of homologous membrane recognition to DI-ZM internalization, a competitive inhibition assay was performed using free HepG2 cell membranes as blocking agents (Figure S5). Pre-incubation with free membranes reduced the intracellular fluorescence intensity of DI-ZM at both 4 h and 6 h compared with the non-blocked group, indicating that the available homologous recognition sites on HepG2 cells were partially occupied by the free membranes. This competitive reduction in DI-ZM uptake further supports that the enhanced internalization of DI-ZM is at least partly mediated by homologous membrane-associated interactions rather than nonspecific nanoparticle uptake alone.

### 3.6. In Vitro Cytotoxicity

MTT assays demonstrated that DI-ZM + L group induced the strongest cytotoxic effect among all treatment groups (Figure 4E), indicating the effective combination of DHA-mediated chemotherapy and ICG-based phototherapy. Both DI-Z and DI-ZM exhibited concentration-dependent cytotoxicity toward HepG2 cells. In addition, laser irradiation further reduced cell viability in ICG-containing groups, confirming the therapeutic contribution of photothermal treatment. To further evaluate the biosafety of the carrier system, the cytotoxicity of blank ZIF-8 was examined at the corresponding concentrations. As shown in Figure S6C, ZIF-8 exhibited negligible cytotoxicity, suggesting that the enhanced cell-killing effect of DI-ZM + L was mainly attributed to the loaded therapeutic agents rather than the carrier itself. These results also indicate the potential biosafety of the nanoparticle system for subsequent in vivo applications.

### 3.7. Tumor Spheroid Penetration

The penetration behavior of the nanoparticles was further evaluated using multicellular tumor spheroids. As shown in Figure 4F, DI-ZM exhibited significantly stronger fluorescence signals within the spheroids than free ICG and DI-Z. Fluorescence intensity analysis (Figure 4H) further confirmed the enhanced accumulation and penetration of DI-ZM in the tumor spheroids. In addition, three-dimensional reconstruction images (Figure 4G) showed markedly stronger fluorescence signals for DI-ZM throughout the spheroid structure. These results suggest that DI-ZM exhibits enhanced penetration behavior in tumor spheroids, which may be associated with improved cellular uptake mediated by biomimetic membrane coating.

### 3.8. Live/Dead Staining

**Figure 5:**
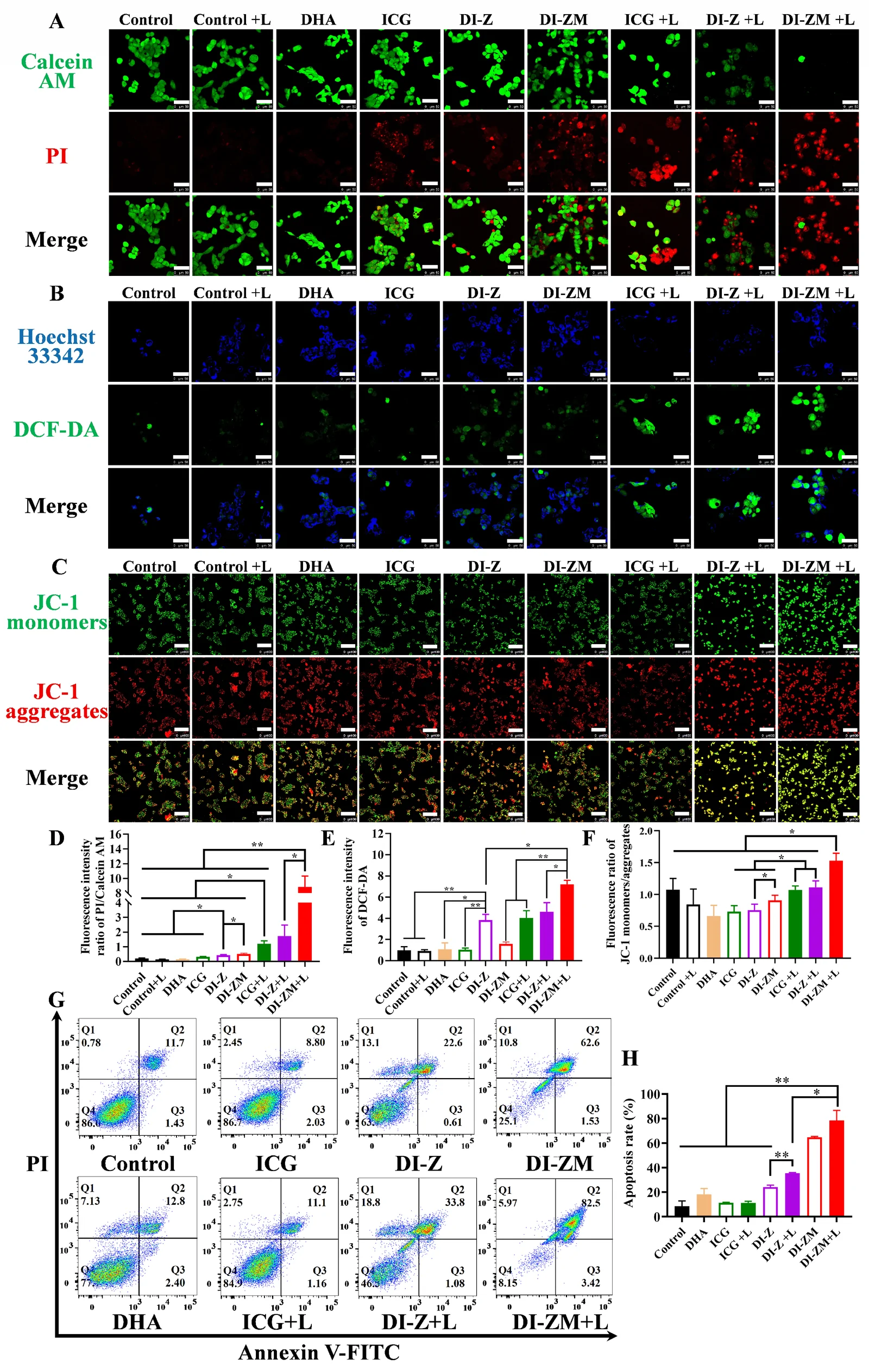
(**A**) CLSM images of live/dead staining in HepG2 cells after different treatments. Scale bars: 50 μm. (**B**) CLSM images of intracellular ROS generation in HepG2 cells detected by DCFH-DA staining. Scale bars: 50 μm. (**C**) CLSM images of mitochondrial membrane potential evaluated by JC-1 staining in HepG2 cells. Scale bars: 100 μm. (**D**) Semi-quantitative fluorescence analysis of live/dead staining, (**E**) intracellular ROS generation, and (**F**) JC-1 red/green fluorescence ratio. (**G**) Flow cytometry analysis of apoptosis in HepG2 cells after different treatments and (**H**) corresponding apoptotic rates. * *p* < 0.05, ** *p* < 0.01.

To assess the therapeutic efficacy of DI-ZM, live/dead staining was performed. As shown in Figure 5A, DI-ZM with laser irradiation induced the strongest red fluorescence signal and the weakest green fluorescence signal among all groups, indicating pronounced cell death. In comparison, the free ICG + L and DI-Z + L groups exhibited moderate red fluorescence signals, whereas only mild cell death was observed in the non-irradiated groups. Semi-quantitative fluorescence analysis (Figure 5D) further confirmed the significantly improved therapeutic efficacy of DI-ZM + L compared to the other treatment groups. These results are consistent with the MTT assay, further demonstrating the superior cell-killing ability of DI-ZM under laser irradiation.

### 3.9. Intracellular ROS Generation

DCFH-DA staining was employed to visualize intracellular ROS production [47]. As shown in Figure 5B, DI-ZM + L displayed the strongest intracellular ROS fluorescence, whereas moderate fluorescence signals were observed in the free ICG + L and DI-Z + L groups. In contrast, only weak ROS fluorescence was detected in the non-irradiated groups. The corresponding semi-quantitative analysis (Figure 5E) suggested that DI-ZM + L generated significantly higher intracellular ROS levels than the other treatments, which may be associated with the enhanced cellular internalization induced by biomimetic membrane coating as well as the combined effects of DHA and ICG. To further quantify intracellular ROS generation, flow cytometry analysis was performed based on DCF fluorescence intensity. Consistent with the CLSM observations, DI-ZM + L induced a clear shift toward higher fluorescence intensity and showed the highest mean fluorescence intensity (MFI), reaching approximately 1.0 × 10^5^ (Figure S7). In comparison, the MFI values of DI-Z + L and DI-ZM were approximately 6.0 × 10^4^, whereas those of the other groups remained below 5.0 × 10^4^. These quantitative results further confirmed that DI-ZM generated the highest intracellular ROS level under laser irradiation. Together, these results indicate that DI-ZM promotes intracellular ROS generation under laser irradiation, which may contribute to its enhanced cancer cell-killing effect.

### 3.10. Mitochondrial Dysfunction and Apoptosis Analysis

Mitochondrial membrane potential was evaluated by JC-1 staining. JC-1 aggregates emit red fluorescence in cells with intact mitochondrial membrane potential, whereas JC-1 monomers show green fluorescence when mitochondrial depolarization occurs [48]. As shown in Figure 5C, DI-ZM + L exhibited the most pronounced increase in green fluorescence accompanied by a marked reduction in red fluorescence, indicating significant mitochondrial membrane depolarization. Consistently, the laser-irradiated groups also exhibited stronger fluorescence changes than the corresponding non-irradiated groups. The corresponding semi-quantitative analysis (Figure 5F) further demonstrated the greatest loss of mitochondrial membrane potential in the DI-ZM + L group. These results suggest that DI-ZM effectively induces mitochondrial dysfunction, which may be associated with excessive intracellular ROS accumulation together with the photothermal effect.

Apoptosis was further analyzed by flow cytometry using Annexin V-FITC/PI staining. As shown in Figure 5G,H, DI-ZM + L induced the highest apoptosis rate (85.9%) with significant differences compared to the other treatment groups. Notably, DI-ZM without laser irradiation also induced measurable apoptosis, reflecting the therapeutic contribution of DHA-mediated chemotherapy. These findings indicate that the combined therapeutic effects of DHA and ICG effectively enhance apoptosis in cancer cells.

### 3.11. Hemocompatibility Evaluation

**Figure 6:**
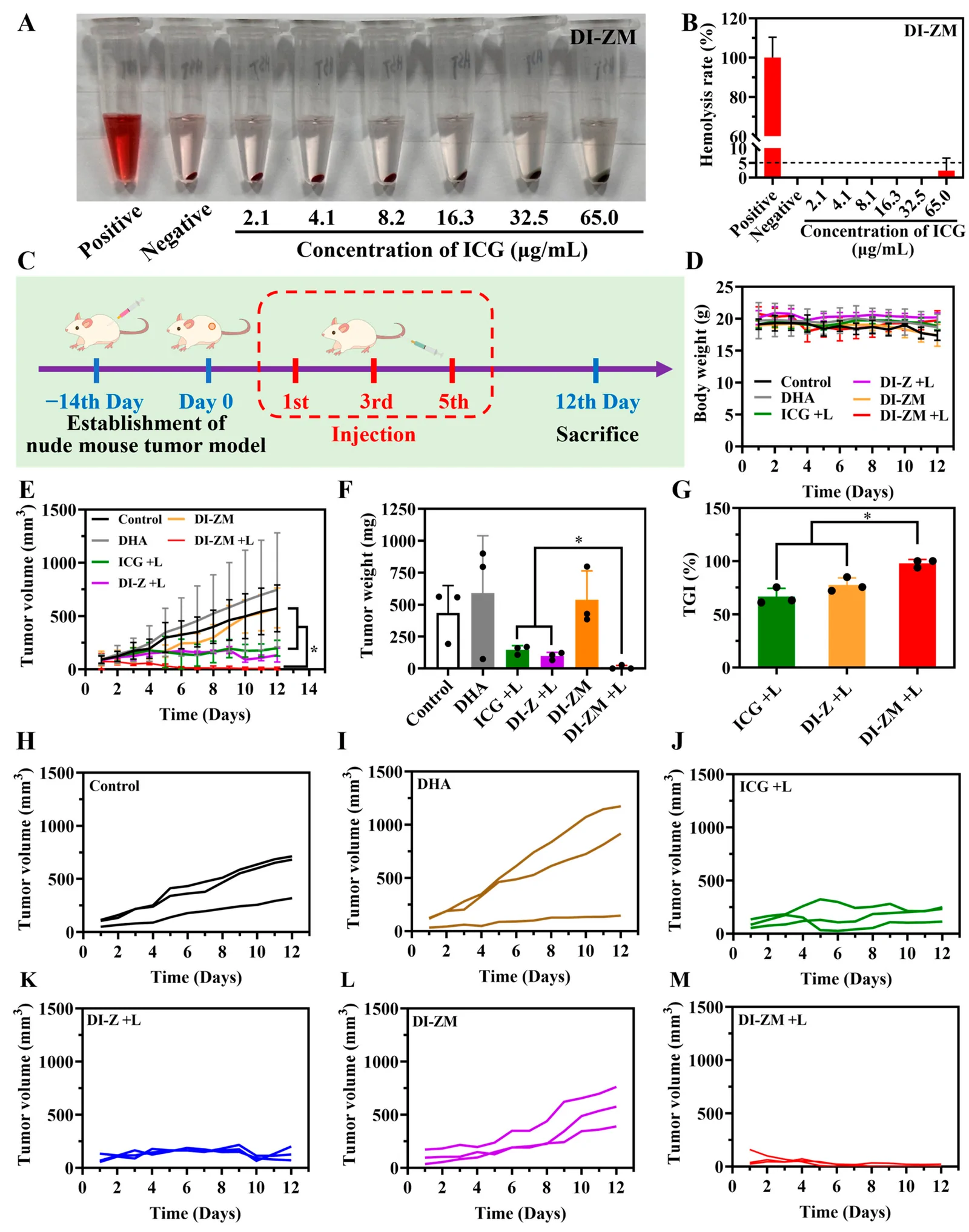
(**A**) Hemolysis photographs of DI-ZM at different concentrations and (**B**) corresponding hemolysis rates (n = 3). (**C**) Schematic illustration of the treatment schedule for HepG2 tumor-bearing mice (n = 3). (**D**,**E**) Body weight changes and tumor growth curves during treatment. (**F**) Tumor weights and (**G**) tumor inhibition rates at the end of treatment (n = 3). (**H**–**M**) Individual tumor growth curves of mice in different treatment groups. * *p* < 0.05.

Good hemocompatibility is essential for the systemic administration of nanomedicines, and hemolysis evaluation provides a direct indication of whether a formulation may disrupt erythrocyte membranes and induce hemoglobin leakage [49]. Therefore, the hemolytic activity of DI-ZM was evaluated using red blood cells. As shown in Figure 6A,B, DI-ZM exhibited negligible hemolysis across all tested concentrations, with hemolysis rates remaining below 5%. The low hemolysis rates observed for DI-ZM suggest that the nanoplatform did not cause obvious erythrocyte membrane damage under the tested conditions. This result may be associated with the biomimetic membrane coating and good colloidal dispersibility of DI-ZM, which could help reduce direct disruptive interactions between the nanoparticle surface and red blood cells. These results indicate favorable blood compatibility and suggest the potential suitability of DI-ZM for intravenous administration.

### 3.12. In Vivo Antitumor Efficacy

The in vivo therapeutic performance was evaluated in a HepG2 tumor-bearing mouse model. Minor body weight fluctuations were observed during the treatment period, but no obvious abnormal weight loss was detected among the different groups (Figure 6D), suggesting acceptable systemic tolerance under the applied therapeutic regimen (Figure 6C). As shown in Figure 6E, DI-ZM + L exhibited the most significant tumor growth inhibition among all groups, with tumor progression markedly suppressed after the first treatment cycle and sustained inhibition maintained throughout the study. In contrast, free DHA showed limited therapeutic efficacy, while free ICG-mediated photothermal treatment achieved only partial tumor suppression. Although DI-ZM without laser irradiation initially delayed tumor growth, rapid tumor regrowth was observed after treatment cessation, indicating the limited efficacy of chemotherapy alone. This phenomenon may be primarily attributed to the pharmacokinetic limitations of DHA, including its relatively rapid systemic clearance and insufficient sustained retention within tumor tissues after treatment cessation [5]. At the end of treatment, tumor weight analysis further supported the tumor volume results (Figure 6F). Partial tumor ablation was observed in the DI-ZM + L group, and the mean weight of the remaining tumors was only 27 mg, markedly lower than that of the control group (437 mg). DI-ZM + L achieved the highest tumor growth inhibition rate (TGI, 97.93 ± 3.59%), demonstrating the superior therapeutic efficacy of our biomimetic nanoplatform (Figure 6G). Consistently, the individual tumor growth curves showed a clear inhibitory trend in the DI-ZM + L group, further supporting the group-averaged tumor volume and TGI results (Figure 6H–M.). The enhanced antitumor performance may be attributed to improved intracellular delivery, enhanced tumor penetration, and the combined chemo-photothermal and photodynamic therapeutic effects.

## 4. Conclusions

In summary, a biomimetic multifunctional nanoplatform (DI-ZM) was successfully developed through the co-loading of DHA and ICG into ZIF-8 nanoparticles followed by HepG2 cell membrane coating. The obtained DI-ZM nanoparticles exhibited favorable colloidal stability, efficient photothermal performance, and effective ROS generation capability under laser irradiation. Benefiting from biomimetic membrane coating, DI-ZM demonstrated enhanced homologous targeting ability, improved cellular internalization, and superior tumor spheroid penetration compared with non-coated nanoparticles. In vitro studies revealed that DI-ZM with laser irradiation effectively promoted intracellular ROS accumulation, mitochondrial membrane depolarization, and apoptosis, with apoptosis rates exceeding 80%, resulting in enhanced cancer cell-killing effects through the combined therapeutic actions of DHA-mediated chemotherapy and ICG-based phototherapy. In vivo studies further demonstrated that DI-ZM with laser irradiation achieved the strongest tumor suppression in HepG2 tumor-bearing mice, with a tumor inhibition rate of 97.93 ± 3.59%, while maintaining acceptable systemic tolerance, demonstrating the superior therapeutic efficacy of the biomimetic nanoplatform. Overall, the enhanced antitumor performance of DI-ZM may be attributed to improved intracellular delivery, enhanced tumor penetration, and the combined chemo-photothermal and photodynamic therapeutic effects. This work highlights the potential of biomimetic membrane-coated nanoplatforms for efficient HCC therapy and provides a promising strategy for the development of multifunctional nanomedicines for combinational cancer treatment.

## Data Availability

The data presented in this study are available on request from the corresponding author.

## Supplementary Materials

The following supporting information can be downloaded at https://www.mdpi.com/article/10.3390/pharmaceutics18081000/s1, Figure S1: The zeta-potential of (A) DI-Z and (B) DI-ZM. Data are shown as mean ± standard error; Figure S2: Hydrodynamic size stability of DI-ZM in serum-containing medium over 7 days; Figure S3: pH-responsive release behavior of ICG from DI-ZM in different pH condition (7.4, 6.4, and 5.4); Figure S4: Evaluation of photothermal properties. (A,B) Temperature elevation curves of ICG at different concentrations and laser power. (C) Photothermal conversion efficiency of ICG. (D–E) Temperature elevation curves and photothermal conversion efficiency of DI-Z at different concentrations and laser power. (F) Photothermal conversion efficiency of DI-Z; Figure S5: CLSM images of DI-ZM uptake in the homologous targeting competition assay. (A) DI-ZM group without pre-incubation with free HepG2 cell membranes. (B) DI-ZM + M group with pre-incubation with free HepG2 cell membranes. Scale bars: 25 μm; Figure S6: Cell viability of HepG2 cells after incubation with (A) free ICG and ICG@ZIF-8, (B) free DHA and DHA@ZIF-8, and (C) ZIF-8 for 24 h, evaluated by MTT assay. Data are presented as mean ± standard error (n = 3). * *p* < 0.05, ** *p* < 0.01, *** *p* < 0.001; Figure S7: (A) Flow cytometric analysis of intracellular ROS generation in HepG2 cells after treatment with PBS, free DHA, free ICG (+/-L), DI-Z (+/-L), or DI-ZM (+/-L), and (B) the corresponding quantitative results. * *p* < 0.05, ** *p* < 0.01, and *** *p* < 0.001; Figure S8: Effect of ROS scavenging on DI-ZM-induced intracellular ROS generation and cytotoxicity in HepG2 cells. (A) Representative CLSM images of intracellular ROS in HepG2 cells treated with PBS, DI-ZM+L, DI-ZM+NAC+L, or DI-ZM+VC+L. DI-ZM was administered at an ICG-equivalent concentration of 8 μg/mL, with NAC and vitamin C used at 20 and 10 mM, respectively. After 4 h, the cells were stained with DCFH-DA and irradiated with laser (0.75 W/cm2, 5 min). Intracellular ROS and nuclei are shown in green and blue, respectively. Scale bar: 25 μm. (B) Viability of HepG2 cells treated with DI-ZM alone or together with NAC (20 mM) or VcNa (10 mM) at ICG-equivalent concentrations of 6, 8, 12, 18, and 22 μg/mL, followed by irradiation (0.25 W/cm^2^, 5 min). Cell viability was determined by the MTT assay after a total treatment period of 24 h. n = 3. * *p* < 0.05, ** *p* < 0.01, and *** *p* < 0.001; Figure S9: Body weight changes of healthy mice after intravenous administration of PBS, free DHA, ICG, DI-Z, or DI-ZM over 5 days (n = 3); Figure S10: Hematological analysis of healthy mice 5 days after intravenous administration of PBS, free DHA/ICG, DI-Z, or DI-ZM (n = 3). (A) Total white blood cell (WBC) count. (B) Neutrophil count (Gran#). (C) Red blood cell (RBC) count. (D) Hemoglobin (HGB) level. (E) Hematocrit (HCT). (F) Mean corpuscular hemoglobin (MCH). (G) Mean corpuscular volume (MCV). (H) Mean corpuscular hemoglobin concentration (MCHC). (I) Platelet (PLT) count levels in each group. * *p* < 0.05, ** *p* < 0.01, and *** *p* < 0.001; Figure S11: Histological and immunostaining analyses of tumor tissues from mice after different treatments. (A) H&E staining, (B) Ki-67 immunostaining, and (C) TUNEL staining of tumor sections. Scale bars: 50 μm. Reference [50] is mentioned in Supplementary Materials file.
