# Supplementary material for: Biomimetic ZIF-8 Nanoplatform for Enhanced Therapeutic Efficacy of Combined Phototherapy and Chemotherapy Against Hepatocellular Carcinoma
Source: Pharmaceutics. 2026 Aug 13;18(8):1000. doi: 10.3390/pharmaceutics18081000 (PMC13516517; doi:10.3390/pharmaceutics18081000)
Supplement: Supplementary file 1 [file pharmaceutics-18-01000-s001.zip › pharmaceutics-4444524-supplementary.pdf]

Supporting Information

Biomimetic ZIF-8 Nanoplatform for Enhanced Therapeutic Efficacy of Combined Phototherapy and Chemotherapy Against Hepatocellular Carcinoma

Materials

Dihydroartemisinin (DHA), dimethyl sulfoxide (DMSO), and ethyl alcohol were obtained from Aladdin (Shanghai, China). Zinc nitrate (Sigma-Aldrich, Merck Life Science, Shanghai, China). 2-methylimidazole and indocyanine green (ICG) were obtained from J&K Scientific (Beijing, China). 1,3-diphenylisobenzofuran and N-Acetyl cysteine (NAC) were purchased from Titan Scientific Co., Ltd. (Adamas-beta, Shanghai, China). MEM medium (Pricella Life Science & Technology Co., Ltd., Wuhan, China). 3-(4,5-Dimethylthiazol-2-yl)-2,5-diphenyltetrazolium bromide (MTT), 4',6-diamidino-2-phenylindole (DAPI), 2',7'-dichlorofluorescin diacetate (DCFH-DA), Calcein-AM/propidium iodide (PI) cell viability/cytotoxicity assay kit, and Hoechst 33342 were purchased from Beyotime Biotechnological Company (Shanghai, China). Sodium ascorbate (VcNa) was purchased from Aladdin Co., Ltd. (Shanghai, China).

The following instruments were used in this study. DLS (Litesizer 500, Anton Paar), TEM (Talos F200S G2, FEI), UV–vis (UV-2600, Shimadzu), CLSM (TCS SP5/SP8/Stellaris 8 FALCON, Leica), microplate reader (Epoch, BioTek), and flow cytometer (FACS Canto™ II, BD) were used for characterization.

SDS–PAGE Analysis

Protein profiles of HepG2 cell membranes, DI-Z, and DI-ZM were analyzed by SDS–PAGE using a commercial kit (Beyotime Biotechnological Company, Shanghai, China) to assess the presence of membrane proteins on the nanoparticle surface. Equal amounts of protein were loaded into each lane. Electrophoresis was performed at 80 V for stacking and 120 V for separation. Gels were stained with Coomassie Brilliant Blue, destained, and imaged using a gel imaging system (Bio Rad, California, USA).

Photothermal Performance Evaluation

Free ICG, DI-Z, and DI-ZM (the ICG concentration of 100 μg/mL, 200 μL) were irradiated (808 nm, 0.5 W/cm^2, 5 min, 10 mm in diameter) and then allowed to cool naturally, with pure water as a control. Temperature profiles were recorded to determine the photothermal conversion efficiency [1]. For stability assessment, free ICG, DI-Z, and DI-ZM (the ICG concentration of 100 μg/mL) were subjected to four cycles of irradiation (808 nm, 0.75 W/cm^2, 5 min) followed by natural cooling to room temperature. Temperature changes were recorded every 30 secs using an infrared thermal imaging camera (Teledyne FLIR, Inc., Oregon, USA). In addition, the effects of concentration, laser power, and irradiation duration on the photothermal heating performance were systematically investigated.

In Vitro ROS Generation

ROS generation was monitored using 1,3-diphenylisobenzofuran (DPBF) as an indicator. DI-Z and DI-ZM were incubated with DPBF and subjected to 808 nm laser irradiation under different conditions. For concentration-dependent evaluation, samples were prepared at ICG-equivalent concentrations ranging from 0 to 10 μg/mL and irradiated (0.75 W/cm^2, 5 min). To assess the effect of laser power, samples containing 5 μg/mL ICG were exposed to different power densities (0–1.5 W/cm^2) for 5 min. The decrease in DPBF absorbance at 417 nm was recorded to evaluate ROS generation.

In Vitro Drug Release

DI-ZM nanoparticles were dispersed in PBS (pH 5.4, 6.4 or 7.4) and incubated at 37ºC under gentle shaking. For ICG release, at predetermined time points (3, 6, 12, 18, 24 h), samples were centrifuged (8000 rpm, 4ºC, 10 min), and 100 μL of supernatant was collected to determine drug release by UV–vis spectroscopy, from which cumulative release was calculated accordingly. For DHA release, DI-ZM nanoparticles were similarly incubated in PBS at different pH values (5.4, 6.4, or 7.4). At predetermined time points (1, 2, 3, 6, 9, 12, 18, and 24 h), samples were centrifuged (3000 rpm, 4 °C, 5 min), and the DHA precipitates were collected and dissolved in 100 μL of ethanol. The released DHA was then quantified by UV-vis spectroscopy, and the cumulative DHA release was calculated accordingly. The cumulative release rate was calculated by the following equation:

$$E\ (\%) = \left(\frac{V_E \sum_1^{n-1} C_i + V_0 C_n}{m_0}\right) \times 100\% \qquad (1)$$

In this equation, E represents the cumulative release amount (%), V_E represents the sampling volume, V_0 represents the initial volume, C_i and C_n represent the measured drug concentrations (μg/mL), i and n represent the sampling times and m_0 represents the initial mass of drug (μg).

Cell Viability Assay

HepG2 cells (5.0 × 10^3 cells per well) were seeded into 96-well plates and incubated overnight. After removal of the culture medium, cells were treated with DI-ZM, DI-Z, DHA@ZIF-8, ICG@ZIF-8, DHA, ICG, or ZIF-8. For laser irradiation groups (+L), cells were irradiated (808 nm, 0.75 W/cm^2, 5 min) after 4 h of incubation. Following a total incubation period of 24 h, the medium was removed and cells were washed with PBS. Subsequently, MTT solution (500 μg/mL, 100 μL) was added to each well and incubated for 4 h. The MTT-containing medium was discarded, and the resultingformazan crystals were dissolved-in 150 μL of dimethyl sulfoxide. The absorbance at 492 nm was measured using a microplate reader, and the cell viability was calculated accordingly [2].

Intracellular ROS Detection

HepG2 cells were seeded in glass-bottom dishes (2 × 10^5 cells per dish) and incubated overnight. Cells were then incubated with free DHA, free ICG (+/-L), DI-Z (+/-L), or DI-ZM (+/-L), at DHA- and ICG-equivalent concentrations (6 and 8 μg/mL), with PBS (+/-L) as the controls, for 4 h. The culture medium was subsequently replaced with fresh medium containing DCFH-DA, followed by incubation at 37°C for 20 min. After washing to remove excess probe, laser irradiation (808 nm, 0.75 W/cm^2, 5 min) was applied to the irradiation groups (+L). The cells were then stained with Hoechst 33342, washed, and observed by CLSM to evaluate intracellular ROS levels. DCF, with the maximum excitation wavelength of 488 nm and the maximum emission wavelength of 525 nm; Hoechst 33342, with the maximum excitation wavelength of 350 nm and the maximum emission wavelength of 461 nm.

For flow cytometric quantification of intracellular ROS, HepG2 cells were seeded in 6-well plates (1 × 10^5 cells per well) and treated under the same conditions as described above. After DCFH-DA staining and laser irradiation, the cells were trypsinized, collected by centrifugation, and resuspended in PBS. The DCF fluorescence intensity was then quantified using a flow cytometer to further assess intracellular ROS generation.

Homologous Targeting Competition Assay

HepG2 cells were seeded in glass-bottom dishes (1 × 10^5 cells per dish) and incubated overnight. To evaluate homologous targeting competition, cells assigned to the DI-ZM + M group were pre-incubated with free HepG2 cell membranes at 37°C for 1 h to competitively interfere with homologous membrane-mediated interactions. Cells in the DI-ZM group were processed in parallel without membrane pre-incubation. Subsequently, both groups were incubated with DI-ZM (equivalent ICG concentration: 4 μg/mL) for 4 or 6 h. After incubation, the cells were fixed with 5% paraformaldehyde for 15 min, stained with DAPI for 5 min, and washed with PBS. Intracellular

fluorescence signals were then observed by CLSM.

Live/Dead Cell Staining

HepG2 cells (2.0 × 10^5 cells per dish) were seeded in glass-bottom dishes and incubated for 12 h. Cells were then treated with free DHA, free ICG (+/-L), DI-Z (+/-L), or DI-ZM (+/-L), at DHA- and ICG-equivalent concentrations (6 and 8 μg/mL), with PBS (+/-L) as the controls. After 4 h, cells in the irradiation groups (+L) were irradiated (808 nm, 0.75 W/cm^2, 5 min). After incubated for another 12 h, cells were washed with PBS and incubated with Calcein-AM/PI staining solution at 37°C for 30 min. Cells were then washed, and fluorescence images were acquired using CLSM to assess cell viability. Calcein, with an excitation maximum at 494 nm and emission maximum at 517 nm. PI-DNA complex, with an excitation maximum at 535 nm and emission maximum at 617 nm.

Mitochondrial Membrane Potential Assay

HepG2 cells were seeded in glass-bottom dishes at a density of 2.5 × 10^5 cells per dish and cultured for 12 h. Cells were then incubated with PBS (+/-L), free DHA, free ICG (+/-L), DI-Z (+/-L), or DI-ZM (+/-L), at equivalent concentrations of DHA (6 μg/mL) and ICG (8 μg/mL) for a total of 24 h. Cells in the irradiation groups (+L) were exposed to a laser (0.75 W/cm^2, 5 min) at 4 h post-treatment. Cells were then washed and stained with JC-1 working solution at 37°C for 20 min. Cells were washed twice with buffer and imaged via CLSM to assess mitochondrial membrane potential. JC-1 polymer, with the maximum excitation wavelength of 525 nm and the maximum emission wavelength of 590 nm; JC-1 monomer, with the maximum excitation wavelength of 490 nm and the maximum emission wavelength of 530 nm.

Hemolysis Assay

Fresh red blood cells were isolated, washed, and diluted to a 2% suspension. The suspension was incubated with DI-ZM at different concentrations for 2 h at 37°C. 1%Triton X-100 was used as the positive control group, while PBS- was used as thenegative control group. Both were incubated with the red-blood cell suspension. After centrifugation (12000 rpm, 25°C, 15 min), photographs were taken, and the absorbance of the supernatant was measured at 545 nm to evaluate hemolysis via microplate reader (BioTek Instruments, Inc., USA). The hemolysis ratio was determined via the following equation:

$$\text{Hemolysis rate (\%)} = \frac{A_{Experiment}-A_{Negative}}{A_{Positive}-A_{Negative}} \times 100\% \quad (2)$$

Where $A_{Experiment}$ represents the absorbance of the sample, $A_{Negative}$ represents the absorbance of the negative control, and $A_{Positive}$ represents the absorbance of the positive

control.

Intracellular ROS Scavenging Assay

HepG2 cells were seeded in glass-bottom dishes at a density of 2×10^5 cells per dish and incubated overnight. The cells were pretreated for 4 h with PBS (control), DI-ZM (DI-ZM+L), DI-ZM supplemented with 20 mM NAC (DI-ZM + NAC + L), or DI-ZM supplemented with 10 mM sodium ascorbate (DI-ZM + VcNa + L), with the ICG-equivalent concentration of DI-ZM fixed at 8 μg/mL. The media were then replaced with fresh medium containing 0.1% DCFH-DA, followed by incubation at 37°C for 20 min in the dark. After removal of the excess probe, the DI-ZM-treated groups were irradiated with an 808 nm laser at 0.75 W/cm^2 for 5 min. The nuclei were subsequently stained with Hoechst 33342, and intracellular ROS fluorescence was visualized using CLSM.

Effect of ROS Scavenging on DI-ZM-Mediated Cytotoxicity

HepG2 cells were seeded in 96-well plates at a density of 5×10^5 cells per well and incubated overnight. The cells were treated with DI-ZM alone (DI-ZM + L), DI-ZM supplemented with 20 mM NAC (DI-ZM + NAC + L), or DI-ZM supplemented with 10 mM sodium ascorbate (DI-ZM + VcNa + L), at ICG-equivalent concentrations of 6, 8, 12, 18, and 22 μg/mL. Untreated cells served as the control. After incubation for 4 h, the treatment groups were exposed to an 808 nm laser at 0.25 W/cm^2 for 5 min and further incubated until the total treatment period reached 24 h. The culture medium was then removed, and the cells were washed with PBS twice. MTT solution (0.5 mg/mL, 100 μL per well) was added and incubated for 4 h. The MTT-containing medium was discarded, and the resulting formazan crystals were dissolved in 150 μL of dimethyl sulfoxide. Absorbance at 570 nm was measured using a microplate reader, and cell viability was calculated relative to that of the untreated control group.

Biosafety Evaluation

To evaluate the preliminary systemic biosafety of the formulations, healthy mice were randomly divided into different groups and treated with PBS, free DHA, ICG, DI-Z, or DI-ZM (n = 3 per group). All formulations were administered once via intravenous injection at doses equivalent to DHA (1.4 mg/kg) and ICG (2.0 mg/kg). Body weight was monitored daily for 5 days after treatment. On day 5, the mice were sacrificed, and whole blood samples were collected for complete blood count (CBC) analysis to evaluate hematological parameters.

Supplementary data

ROS Scavenging Analysis of DI-ZM-Mediated Therapeutic Effects

ROS generation is an important mechanism involved in ICG-mediated photodynamic effects and may contribute to the enhanced therapeutic efficacy of DI-ZM under laser irradiation. To clarify the involvement of ROS, intracellular ROS levels were examined in the presence of the antioxidants NAC and VcNa. As shown in Figure S8A, DI-ZM + L treatment induced strong intracellular ROS fluorescence in HepG2 cells, whereas pretreatment with NAC or VcNa substantially decreased the ROS signal. This reduction confirmed that the enhanced fluorescence observed after DI-ZM + L treatment was associated with intracellular ROS accumulation and demonstrated the effective antioxidant activity of both scavengers under the experimental conditions. The results support the involvement of ROS in the photodynamic component of DI-ZM-mediated cytotoxicity.

Effect of ROS Scavenging on DI-ZM-Mediated Cytotoxicity

The contribution of ROS to DI-ZM-mediated cytotoxicity was further examined by MTT assay in the presence of NAC or sodium ascorbate (VcNa). As shown in Figure S8B, ROS scavenging resulted in only a modest increase in cell viability at the lower ICG-equivalent concentrations of 6 and 8 μg/mL. In contrast, at 12, 18, and 22 μg/mL, both NAC and VcNa produced a clearer increase in cell viability, and this protective effect became more pronounced as the DI-ZM concentration increased. This concentration-dependent response suggests that ROS-mediated damage makes a greater contribution to DI-ZM+L-induced cytotoxicity at higher formulation concentrations. Nevertheless, neither NAC nor VcNa completely restored cell viability, indicating that ROS is an important but not the sole contributor to the antitumor activity of DI-ZM+L. Other therapeutic mechanisms, including ICG-mediated photothermal effects and DHA-mediated chemotherapy, may also contribute to the overall cytotoxic response.

Biosafety Evaluation

The preliminary systemic biosafety of DI-ZM was further evaluated in healthy mice. As shown in Figure S9, no obvious body weight loss was observed in mice treated with PBS, free DHA, ICG, DI-Z, or DI-ZM during the 5-day observation period, suggesting that the formulations did not induce apparent acute systemic discomfort under the tested dose regimen. Complete blood count (CBC) analysis was further performed on day 5 to assess potential hematological effects (Figure S10). Compared with the PBS control group, the DI-ZM-treated mice showed no marked abnormalities in key hematological parameters, including white blood cell (WBC) and red blood cell (RBC) counts. These results indicate that DI-ZM exhibited acceptable preliminary hematological

compatibility after intravenous administration.

Histological and Immunostaining Analysis of Tumor Tissues

Histological and immunostaining analyses were performed on available tumor tissues to provide supplementary pathological evidence of treatment response (Figure S11). Considering the marked tumor regression observed in the DI-ZM+L group, the remaining tumor tissues available for sectioning may mainly represent residual tumor regions rather than the full treatment response. Therefore, the staining results were interpreted as supportive observations. In the available tumor sections, H&E and Ki-67 staining showed only modest differences among the treatment groups, suggesting that these images should be interpreted mainly as supplementary histological observations rather than definitive quantitative evidence. By contrast, TUNEL staining showed more apparent apoptotic signals in the DI-ZM+L group, indicating increased tumor cell apoptosis after treatment. These results provide complementary histological support for the antitumor activity of DI-ZM-mediated chemo-phototherapy.

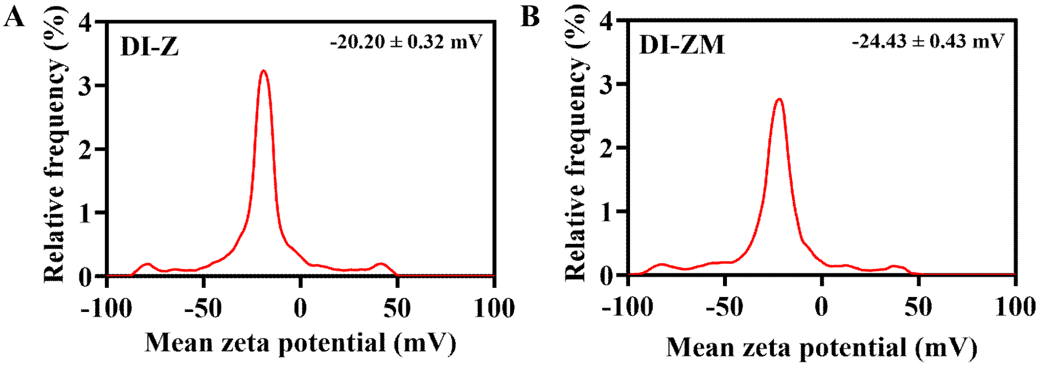

Figure S1. The zeta-potential of (A) DI-Z and (B) DI-ZM. Data are shown as mean ± standard error.

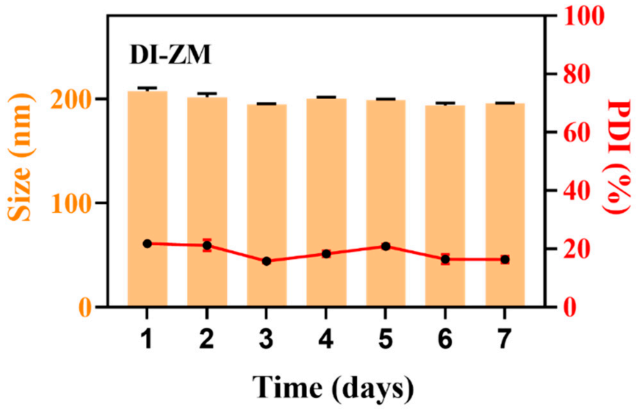

Figure S2. Hydrodynamic size stability of DI-ZM in serum-containing medium over 7 days.

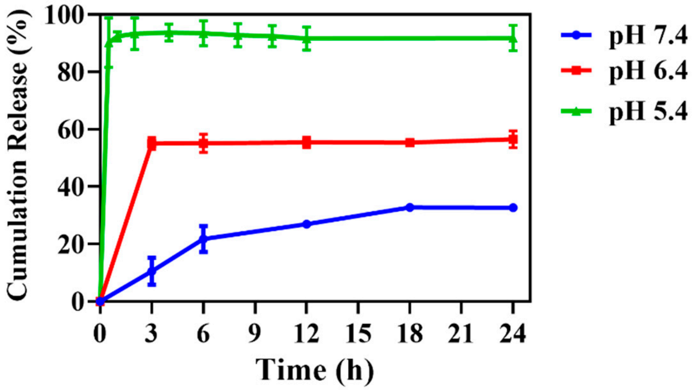

Figure S3. pH-responsive release behavior of ICG from DI-ZM in different pH condition (7.4, 6.4, and 5.4).

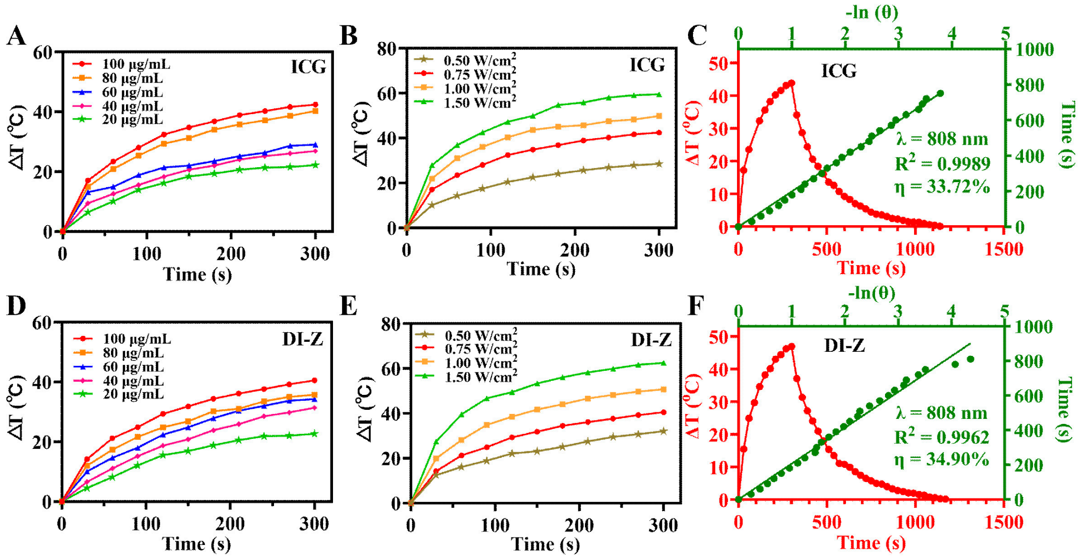

Figure S4. Evaluation of photothermal properties. (A–B) Temperature elevation curves of ICG at different concentrations and laser power. (C) Photothermal conversion efficiency of ICG. (D–E) Temperature elevation curves and photothermal conversion efficiency of DI-Z at different concentrations and laser power. (F) Photothermal conversion efficiency of DI-Z

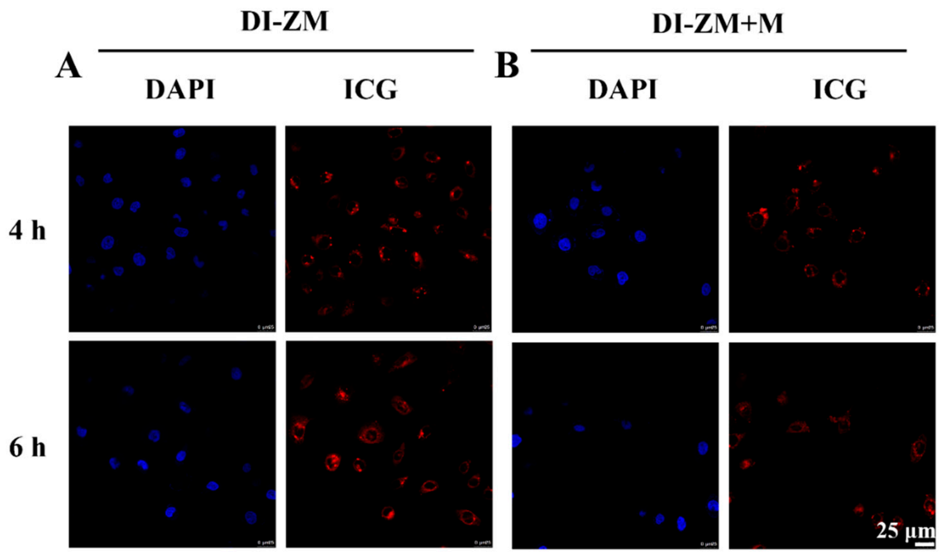

Figure S5. CLSM images of DI-ZM uptake in the homologous targeting competition assay. (A) DI-ZM group without pre-incubation with free HepG2 cell membranes. (B) DI-ZM + M group with pre-incubation with free HepG2 cell membranes. Scale bars: 25 μm

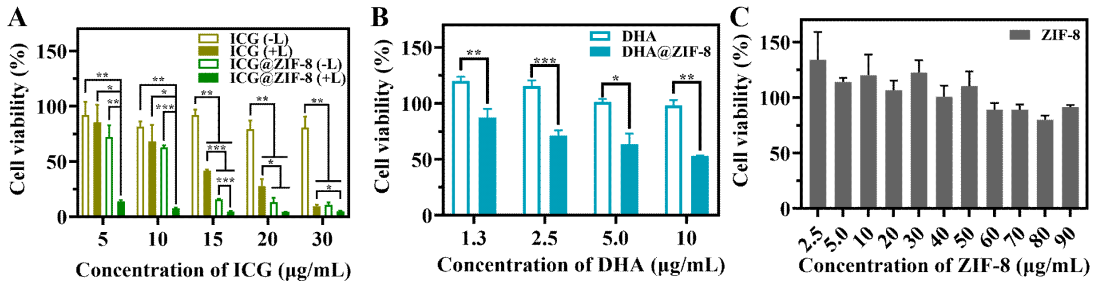

Figure S6. Cell viability of HepG2 cells after incubation with (A) free ICG and ICG@ZIF-8, (B) free DHA and DHA@ZIF-8, and (C) ZIF-8 for 24 h, evaluated by MTT assay. Data are presented as mean ± standard error (n = 3). *$p < 0.05$, **$p < 0.01$, ***$p < 0.001$

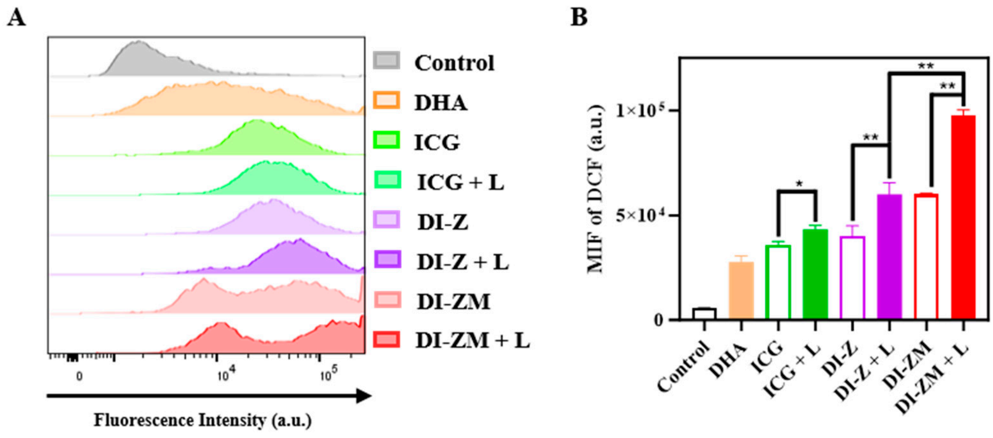

Figure S7. (A) Flow cytometric analysis of intracellular ROS generation in HepG2 cells after treatment with PBS, free DHA, free ICG (+/-L), DI-Z (+/-L), or DI-ZM (+/-

L), and (B) the corresponding quantitative results. *$p < 0.05$, **$p < 0.01$, and ***$p <$ 0.001.

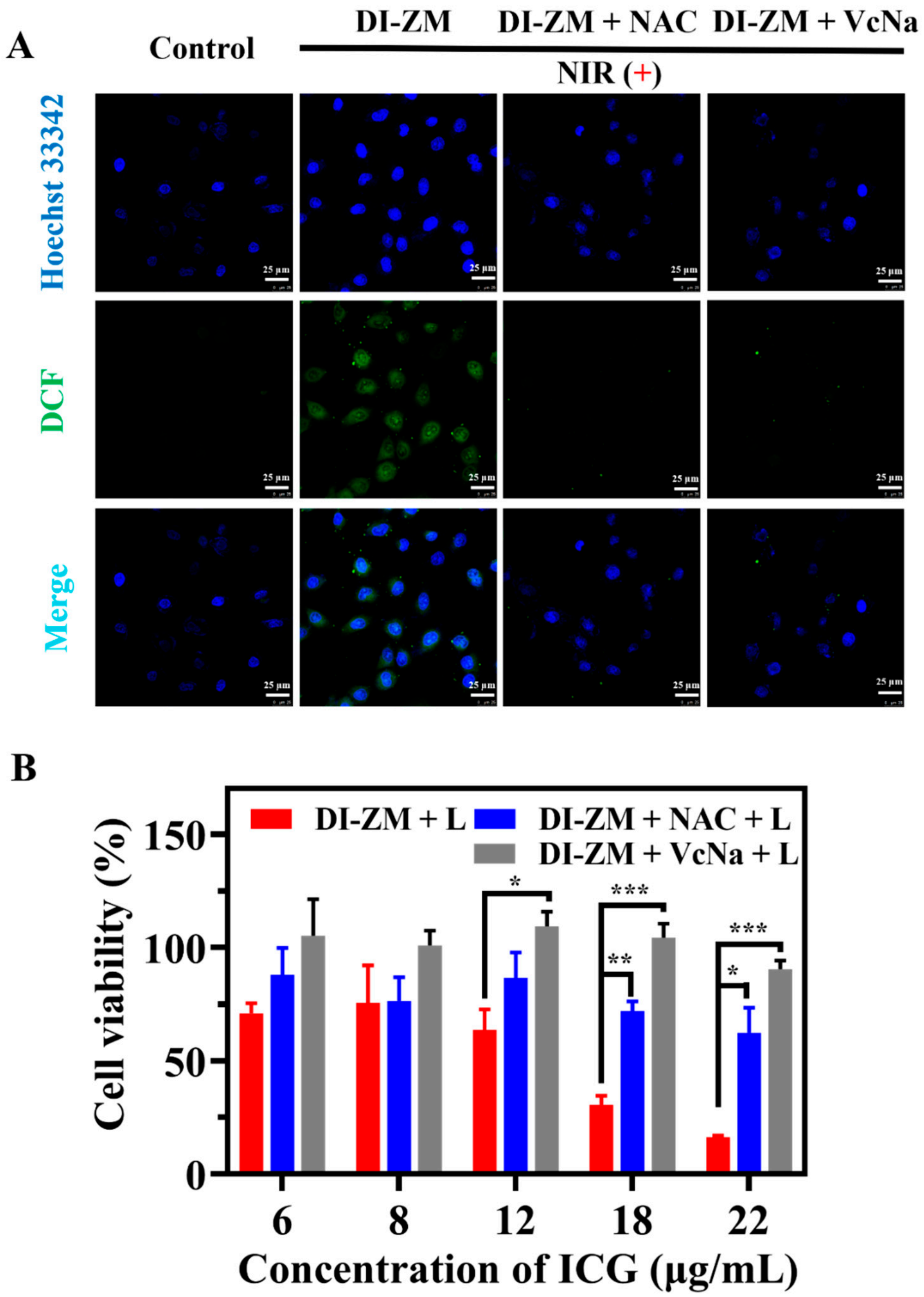

Figure S8. Effect of ROS scavenging on DI-ZM-induced intracellular ROS generation and cytotoxicity in HepG2 cells. (A) Representative CLSM images of intracellular ROS in HepG2 cells treated with PBS, DI-ZM+L, DI-ZM+NAC+L, or DI-ZM+VC+L. DI-ZM was administered at an ICG-equivalent concentration of 8 μg/mL, with NAC and vitamin C used at 20 and 10 mM, respectively. After 4 h, the cells were stained with DCFH-DA and irradiated with laser (0.75 W/cm^2, 5 min). Intracellular ROS and nuclei are shown in green and blue, respectively. Scale bar: 25 μm. (B) Viability of HepG2 cells treated with DI-ZM alone or together with NAC (20 mM) or VcNa (10 mM) at

ICG-equivalent concentrations of 6, 8, 12, 18, and 22 μg/mL, followed by irradiation (0.25 W/cm^2, 5 min). Cell viability was determined by the MTT assay after a total treatment period of 24 h. n = 3. *$p < 0.05$, **$p < 0.01$, and ***$p < 0.001$.

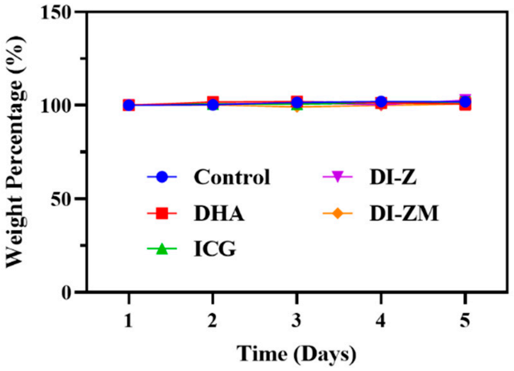

Figure S9. Body weight changes of healthy mice after intravenous administration of PBS, free DHA, ICG, DI-Z, or DI-ZM over 5 days (n = 3).

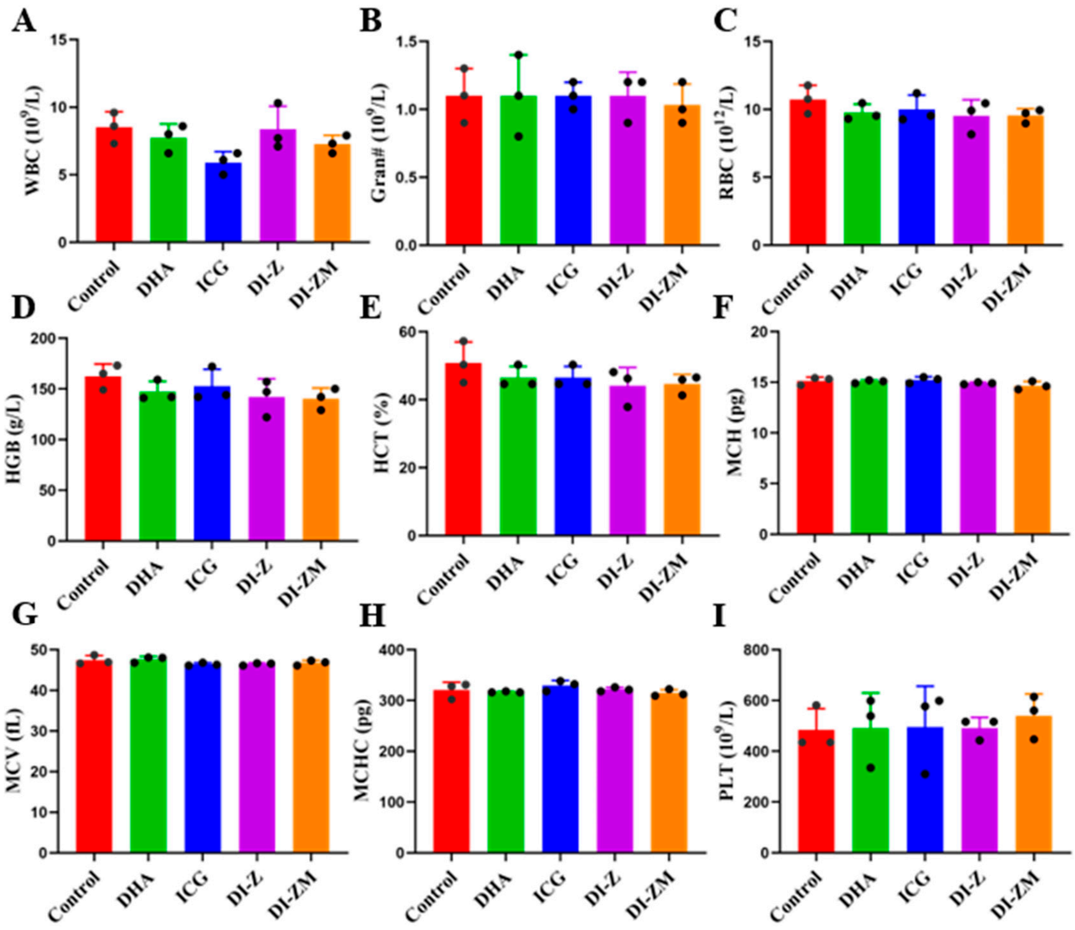

Figure S10. Hematological analysis of healthy mice 5 days after intravenous administration of PBS, free DHA/ICG, DI-Z, or DI-ZM (n = 3). (A) Total white blood cell (WBC) count. (B) Neutrophil count (Gran#). (C) Red blood cell (RBC) count. (D) Hemoglobin (HGB) level. (E) Hematocrit (HCT). (F) Mean corpuscular hemoglobin (MCH). (G) Mean corpuscular volume (MCV). (H) Mean corpuscular hemoglobin

concentration (MCHC). (I) Platelet (PLT) count levels in each group. *$p < 0.05$, **$p < 0.01$, and ***$p < 0.001$.*

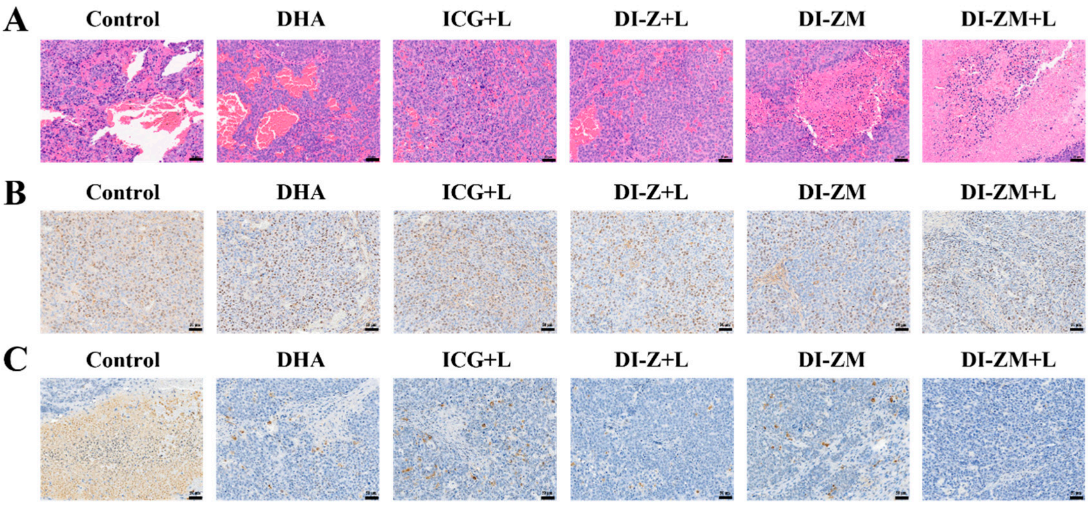

Figure S11. Histological and immunostaining analyses of tumor tissues from mice after different treatments. (A) H&E staining, (B) Ki-67 immunostaining, and (C) TUNEL staining of tumor sections. Scale bars: 50 μm

Reference

[1] He, S.; Zheng, Q.; Ma, L.; Shen, H.; Zheng, B.; Zhang, Y.; Deng, H. H.; Chen, W.; Fan, K. Mucin-triggered osmium nanoclusters as protein-corona-like nanozymes with photothermal-enhanced peroxidase-like activity for tumor-specific therapy. *Nano Lett.*, **2024**, *24*, 14337–14345.

[2] Jia, J.; Chen, W.; Xu, L.; Wang, X.; Li, M.; Wang, B.; Huang, X.; Wang, T.; Chen, Y.; Li, M.; Tian, D.; Zhuang, J.; Lin, X.; Li, N. Codelivery of dihydroartemisinin and chlorin e6 by copolymer nanoparticles enables boosting photodynamic therapy of breast cancer with low-power irradiation. *Regener. Biomater*, **2023**, *10*, rbad048.
